# Plants Can Benefit from Herbivory: Stimulatory Effects of Sheep Saliva on Growth of *Leymus chinensis*

**DOI:** 10.1371/journal.pone.0029259

**Published:** 2012-01-03

**Authors:** Jushan Liu, Ling Wang, Deli Wang, Stephen P. Bonser, Fang Sun, Yifa Zhou, Ying Gao, Xing Teng

**Affiliations:** 1 Key Laboratory of Vegetation Ecology, Ministry of Education, Institute of Grassland Science, Northeast Normal University, Changchun, Peoples Republic of China; 2 Evolution and Ecology Research Centre, School of Biological, Earth and Environmental Sciences, University of New South Wales, Sydney, Australia; Max Planck Institute for Chemical Ecology, Germany

## Abstract

**Background:**

Plants and herbivores can evolve beneficial interactions. Growth factors found in animal saliva are probably key factors underlying plant compensatory responses to herbivory. However, there is still a lack of knowledge about how animal saliva interacts with herbivory intensities and how saliva can mobilize photosynthate reserves in damaged plants.

**Methodology/Principal Findings:**

The study examined compensatory responses to herbivory and sheep saliva addition for the grass species *Leymus chinensis* in three experiments over three years. The first two experiments were conducted in a factorial design with clipping (four levels in 2006 and five in 2007) and two saliva treatment levels. The third experiment examined the mobilization and allocation of stored carbohydrates following clipping and saliva addition treatments. Animal saliva significantly increased tiller number, number of buds, and biomass, however, there was no effect on height. Furthermore, saliva effects were dependent on herbivory intensities, associated with meristem distribution within perennial grass. Animal saliva was found to accelerate hydrolyzation of fructans and accumulation of glucose and fructose.

**Conclusions/Significance:**

The results demonstrated a link between saliva and the mobilization of carbohydrates following herbivory, which is an important advance in our understanding of the evolution of plant responses to herbivory. Herbivory intensity dependence of the effects of saliva stresses the significance of optimal grazing management.

## Introduction

Herbivory can limit the growth and survivorship of plants, and plants have evolved complex responses to avoid being consumed and/or to survive and flourish after herbivory. It is widely accepted that plants can tolerate physical and biotic stresses and damage [1], [2]. Plant compensatory growth is ubiquitous in nature and an important adaptive response to herbivory [3], [4]. There is some experimental evidence that herbivory may stimulate plant growth and increase plant fitness [5], [6], [7]. However, compensation (and overcompensation) responses are not consistent across species or environments. It has been demonstrated that plant response to herbivory is species specific and compensation to herbivory is specific to herbivory type and intensities [8], [9], [10], [11], [12]. So studies are required to establish the environmental cues plants use to initiate a compensation response. Animal saliva may be an important cue plants use to stimulate growth and initiate compensation [13], [14], [15].

Vittoria and Rendina (1960) originally suggested that grazers caused plant growth stimulation by depositing saliva during grazing, and later tests supported this hypothesis [16], [17], [18]. However, there are some studies demonstrating that herbivore saliva had no, or even negative impacts on plants [19], [20], [21], [22] The positive impacts appear possible in view of growth regulators in salivary systems of insects, such as cytokinins, auxins, and jasmonic acid [9], [12], [23], [24], and various growth factors in mammalian submaxillary glands, including thiamine, nerve growth factor (NGF), transforming growth factor (TGF) and epidermal growth factor (EGF) [25]. Growth factors can intervene directly in cellular metabolism by promoting differential transcription of genes, so they may be expected to have activity in a variety of organisms [26]. Jasmonate was found to be involved in tuber size regulation by mediating cell expansion, which was correlated with increased accumulation of sucrose [11], [12]. Thiamine is a plant growth factor produced in shoots that is necessary for root growth [27]. Dyer and Bokhari (1976) reported grasshoppers might inject growth-promoting substance into *Bouteloua gracilis* and stimulated tiller production. Mouse and human EGF were found to enhance plant growth rate and promote cell division of epicotyl [28], [29].

Recent research in woody plants demonstrates that animal saliva tended to stimulate branching [30], [31]. The activation of dormant meristems is crucial for compensatory growth following herbivory, especially for branching in woody plants or tillering in grasses [32]. Responses in these growth forms both arise from outgrowth of axillary meristems after releasing of apical dominance, which is under genetic and hormonal control [33]. On the grassland of Inner Mongolia, Zhang *et al.* (2007) studied the effects of sheep saliva on a semi-shrub and herbaceous species, and found that sheep saliva stimulated tillering of herbaceous grass [34]. Plant response varies with herbivory intensities, where plants tend to perform better under light herbivory intensity [8], [14], [35]. At light herbivory intensity, there is large possibility for plants to overcompensate for tissue loss, and animal saliva may be one of the mechanisms behind overcompensation [13], [14]. Plant regrowth after herbivory depends on the availability and remobilization of carbon reserve, and the availability of reserve meristems to be allocated to new growth [36], [37]. The different availability of carbon and meristem reserve is responsible for the nonlinear response of plant to herbivory intensities [2], [32], [38], [39]. Despite of some research on plant response to animal saliva, the mechanism behind the response remains uncertain. No study has examined the role of animal saliva in inducing plant compensatory growth after herbivory damage, how the effects of saliva vary with herbivory intensities and how saliva affects resource allocation during regrowth after herbivory.

We conducted experiments to test the role of sheep saliva in promoting compensatory responses to herbivory and mobilization of stored resources in the perennial grass *Leymus chinensis*. We hypothesized that: (1) saliva has largest effects at light herbivory intensities, and (2) animal saliva could promote mobilization of stored carbon reserve.

## Methods

### Ethics Statement

No specific permits were required for this study, because the performance of this study was in accordance with guidelines set by the Northeast Normal University. No specific permits or approval was required for the animal work, because the care of sheep in the studies was in accordance with relevant national and international guidelines. To collect saliva, we put a cake of sponge into sheep mouth when they chewed grasses. After about two minutes, the sponge was taken out. All the performance was softly conducted by hand, without any hurt or damage on the animals. No specific permits were required for the described field studies, because the field is owned by Northeast Normal University and the Songnen Grassland Ecological Research Station performs the management. No specific permits were required for these locations/activities, because the location is not privately-owned or protected in any way and the field studies did not involve endangered or protected species.

### Species and sites

We conducted three experiments at the Songnen Grassland Ecological Research Station of Northeast Normal University, Jilin Province, PR China (44°45′N, 123°45′E). There is a semi-arid and continental climate with a frost-free period of about 140 days, with annual mean temperature ranging from 4.6°C to 6.4°C and annual precipitation from 290 to 450 mm. The main vegetation type is meadow steppe predominated by *Leymus chinensis* and *Stipa baicalensis* [40].

*L. chinensis* is a perennial rhizomatous grass with good palatability and high forage value [8], [40], [41], [42], [43], [44]. It is widely distributed in the eastern region of the Eurasian steppe zone as a dominant species from arid to semi-arid steppes in northern China and eastern Mongolia, and it has extensive plasticity in morphological and physiological characteristics. *L. chinensis* is a clonal perennial grass with large belowground bud bank.This species has the capacity of rapid regrowth after grazing or mowing early in the season, and high tolerance to drought, cold and alkali stresses [45], [46]. Highly branched rhizomes lie horizontally about 5–15 cm beneath the soil surface, and the long rhizomes can spread and form near monocultural stands.

### Culture of experimental plants

At the beginning of May 2006, 2007 and 2008, seeds of *L. chinensis* collected from the study area were germinated in bunched paper cylinders (2 cm in diameter, 5 cm deep) which were filled with soil to about 4 cm in depth and covered with 1 cm of soil again after seeds were sprinkled in cylinders. Cylinders were kept in a greenhouse and watered daily. At about 30 days of age, 13 seedlings of similar size plants per pot, were transplanted into outdoor plastic pots (20 cm in diameter and 15.5 cm deep) filled with 14 cm field soil in 2006, and in 2007 with the mixture of field soil and fertile soil from commercial source in a 6∶1 ratio. In the two experiments, grasses were watered daily. In 2008, seedlings were transplanted into pots filled with sand and watered with 1/7 strength Hoagland's solution every day.

### Saliva collection and application

Saliva was collected by inserting a cake of sponge into the mouth of a sheep. The sheep chewed on the sponge for two minutes and the sheep saliva was squeezed into a tube. After being filtered by sponge, saliva was clean and there was no plant material mixed in. For the saliva addition treatment in each of the experiments, we clipped grasses and immediately applied saliva with a mini brush across the cut end of the leaves (clipped plants) or along the length of the leaf blades (non-clipped plants). The sponge, tube and brush were sterilized with 75% alcohol and dried before used.

### Experimental design and measurements

#### Experiment 1, effects on plant growth

We performed the first experiment from 18 July to 18 September, 2006. About one month after being transplanted and adjusted to the outdoor growing conditions, seedlings were assigned to one of four clipping treatments (0, 25, 75 and 100% of above ground shoot height) and one of two levels of saliva (with and without saliva at every clipping level). In another experiment, we studied plant response to different component of animal saliva, which showed that there was no difference between clipping with- and without water (unpublished data). Therefore, in present study we focused on the difference between clipping with- versus without saliva, and there was no clipping with water as control. There were 5 replicate pots per treatment, and plants were harvested one month after treatments. This resulted in a total of 40 pots in the experiment (4 clipping×2 saliva×5 replicates). Plants were randomly assigned to all treatments and 5 µl sheep saliva per seedling was added to saliva treated plants. All treatments were performed within about 2 hours, alternating between the two kinds of treatments at every clipping level (clipping alone versus clipping with saliva) so as to prevent any temporal bias. For every treatment, 10 shoots per pot were randomly marked with wire rings, to measure shoot height.

For nondestructive sampling, we measured the height of the marked shoots and counted the amounts of tillers in all the pots for every treatment on 17 August. After measuring height and tiller number, we harvested the grasses and counted the number of buds. Grasses were separated into above- and belowground parts, and oven dried at 70°C for more than 72 hours prior to measuring biomass. The belowground tissue was carefully washed prior to drying.

#### Experiment 2, effects on plant growth

The second experiment was conducted from 13 July to 20 August 2007. The design was similar to the first one except that there were 5 clipping levels (0, 25, 50, 75 and 100%) and 6 replicates for every treatment combination. The measurements and samplings were also performed one month after treatments (20 August), and there were 60 pots in total.

#### Experiment 3, response in carbohydrate mobilization

Third experiment was conducted from 4 to 14 August, 2008. Thirty six pots of grasses were randomly allocated to two treatments, clipping without saliva, and clipping with saliva. There were three replicates for each treatment. All the plants were clipped at 25% of shoot height. In clipping with saliva treatment, plants were applied with sheep saliva immediately after clipping. After 0, 0.5, 1, 3, 5 and 10 days of regrowth, three pots of plants in every treatment combination were harvested and divided into leaves, stems, rhizomes and fibrous roots, frozen in liquid nitrogen, stored at −80°C and used for analysis of water-soluble carbohydrate.

One hundred milligrams of frozen-dried plant tissue was sampled from harvest plants and ground. A fine powder was boiled in 4 ml 80% ethanol and extracted for 1 hour at 80°C. The sample was centrifuged at 10,000 *g* for 10 min after ethanol extraction, and then the supernatant was preserved. Ten millilitre of water was added to the pellet and the tube contents were mixed and incubated for 1 hour at 90°C. After the aqueous extraction, the sample was centrifuged at 10,000 *g* for 10 min. Then the supernatant was preserved and the aqueous extraction was repeated once again with the pellet. The three supernatants were pooled and evaporated to dryness. The residue was dissolved in 2 ml water, pooled and filtered with a 0.45-µm nylon membrane.

Aliquots of carbohydrate extract were passed through a column containing cation-exchange resin (Dowex 50W X8-400 H^+^-form; Sigma) and a column filled with anion-exchange resin (Amberlite CG-400 II; Fluka) to remove charged compounds. Purified carbohydrates were separated and quantified by high-performance liquid chromatography (HPLC) on a Sugar-PAK column (300 mm long, 6.5 mm i.d.; Millipore Waters), eluted at 0.5 ml min^−1^ and 85°C with 0.1 mM CaEDTA in water, using mannitol as internal standard and a refractometer as a sugar detector [47].

### Statistical analysis

For plant growth variables in Experiment 1 and 2, we performed two-way factorial ANOVA to evaluate the effects of clipping and saliva at every sampling time, with saliva, clipping and their interaction as fixed factors. Tukey-Kramer test was followed to examine the difference among clipping levels. Moreover, Bonferroni correctiont-test was carried out to compare the difference between treatments with- versus without saliva at every clipping level, in whici the “p” value for each test was equal to alpha divided by the number of test (n = 4 in Experiment 1 and n = 5 in Experiment 2). Variables were log transformed, where necessary, to meet the assumptions of statistical analyses. In Experiment 3 to assess the effects of treatments on carbohydrate and how they varied with time, a repeated measures analysis of variance (ANOVA) was also used with clipping without- versus clipping with saliva as between-subject factor (main effect), and time as within-subject (repeated) factor. Bonferroni correction was carried out to analyze the difference between treatments (clipping without- versus with saliva) at every time, and the “p” value was adjusted based on the number of test (n = 6). All statistical analyses were conducted in SAS 9.0 (SAS Institute, Cary, NC, USA).

## Results

### Growth responses to clipping and saliva

#### Shoot elongation

**Figure 1:**
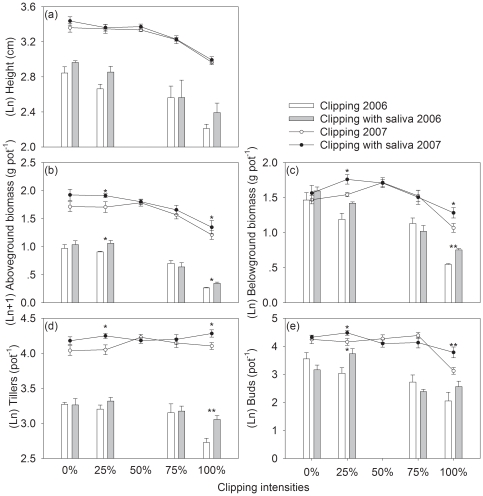
Effects on regrowth. The effects of clipping and saliva on height, aboveground biomass, belowground biomass (BGB), tillers and buds (back-transformed from the log scale) of *Leymus chinensis* both on 17 August 2006 and 20 August 2007. There are four clipping levels (0%, 25%, 75% and 100% of aboveground shoots) in 2006 and five ones (0%, 25%, 50%, 75% and 100%) in 2007. Bars represent standard errors. **, P<0.05; *, P<0.01.

Both in 2006 and 2007, plant height decreased with increasing clipping intensities (Fig. 1, Table 1). In the two experiments, there was neither significant saliva effect nor interactive effect between saliva and clipping (Table 2), although there was a trend towards an increase when saliva was applied to clipped shoots (Fig. 1).

**Table 1:** Results of Duncan multiple comparisons of differences in height, aboveground biomass (AGB), belowground biomass (BGB), tillers and buds (back-transformed from the log scale) among clipping levels both in 2006 and 2007.

		Height	AGB	BGB	Tillers	Buds
2006	0%	2.87a	1.00a	1.58a	3.53a	3.80a
	25%	2.74ab	1.07a	1.60a	3.63a	3.91a
	75%	2.67b	0.89a	1.37a	3.54a	3.59a
	100%	2.32c	0.39b	0.67b	3.27b	2.72b
2007	0%	3.40a	1.82a	1.60ab	4.11a	4.29a
	25%	3.36a	1.81a	1.65ab	4.14a	4.35a
	50%	3.35a	1.79a	1.71a	4.21a	4.19a
	75%	3.23b	1.61b	1.51b	4.17a	4.26a
	100%	2.98c	1.27c	1.17c	4.19a	3.45b

Different letters indicate statistical significance at P<0.05 (n = 5 in 2006, n = 6 in 2007).

#### Accumulation in biomass

In 2006, above- and belowground biomass decreased with increasing clipping intensities, except that plant compensated in above ground biomass at 25% clipping level (Table 1). In 2007, there was no difference among 0%, 25% and 50% clipping treatments, and at 75% and 100% clipping treatments biomass decreased significantly (Table 1). At 25% clipping level, adding saliva on clipped shoots significantly increased aboveground biomass on 2006 and 2007 (Fig. 1). At 100% clipping level, in contrast to clipped plants without saliva, the clipped and saliva-applied grasses produced significantly more above- and belowground biomass in the two years (Fig. 1), and saliva had significant effects on above and belowground biomass (Table 2)

**Table 2:** Results of two ways ANOVA for the effects of saliva, clipping and their interaction on height, aboveground biomass (AGB), belowground biomass (BGB), tillers and buds both in 2006 and 2007.

			Height		AGB		BGB		Tillers		Buds	
Time	Treatments	df	*F*	*P*	*F*	*P*	*F*	*P*	*F*	*P*	*F*	*P*
2006	Saliva	1	3.80	0.0691	1.81	0.1897	2.50	0.1269	4.73	0.0377*	0.29	0.595
	Clipping	3	13.74	0.0001**	64.54	0.0001**	41.67	0.0001**	11.30	0.0001**	13.83	0.0001**
	Saliva×Clipping	3	0.37	0.7740	1.37	0.2736	2.07	0.1309	2.20	0.1090	3.91	0.0177*
2007	Saliva	1	1.70	0.1989	7.96	0.0071**	6.09	0.0179*	7.94	0.0058**	5.62	0.0224*
	Clipping	4	32.45	0.0001**	14.95	0.0001**	16.27	0.0001**	0.97	0.4276	14.54	0.0001**
	Saliva×Clipping	4	0.36	0.8367	0.15	0.9618	1.88	0.1317	1.55	0.1943	2.78	0.0387*

**, *P*<0.05;

*, *P*<0.01.

#### Dynamics of tillering

In experiment 1, the 100% clipping treated plants had significantly fewer tillers than the other clipping treatments, whereas, in experiment 2, there was no difference among clipping treatments (Table 1, 2). In 2006, at 100% clipping level, the clipped and saliva applied plants had significantly more tillers than the grasses clipped without saliva, whereas, in 2007, the overall saliva effect resulted from the significant increase in tillers at 25% and 100% clipping level (Fig. 1). No significantly interactive effect between saliva and clipping was found (Table 2).

#### Changes in the bud bank

In 2006, plants at 0% and 25% clipping levels had significantly more buds than those at 75% and 100% ones (Table 1). In 2007, clipping effects came only from the difference between 100% clipped grasses and those at other clipping levels (Table 1). All the saliva effects and interactive effects with clipping resulted from the difference between clipping with and without saliva at 25% (2006), or at 25% and 100% clipping levels (2007) (Table 1, Fig. 1).

### Carbohydrate mobilization

#### Hydrolyzation of fructans and changes in sucrose

**Figure 2:**
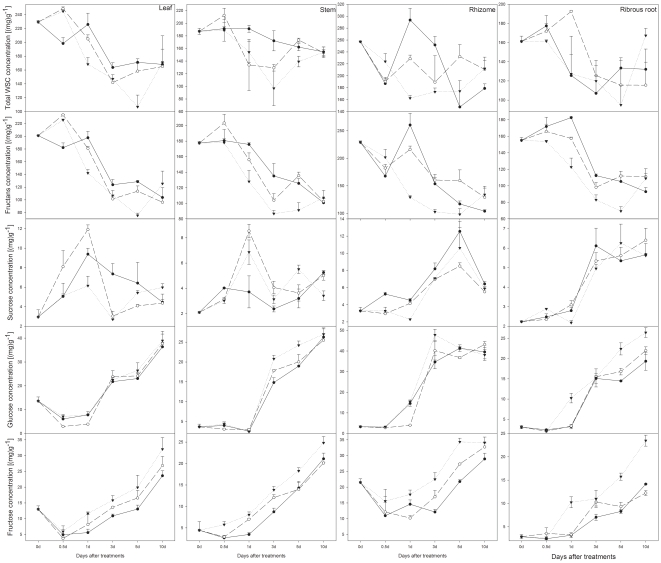
Response of carbohydrate concentrations. The differences between clipping without (real line) and with saliva (broken line) in carbohydrate concentrations, fructans (a_1_–a_4_), sucrose (b_1_–b_4_), glucose (c_1_–c_4_) and fructose (d_1_–d_4_) in component parts, leaf (a_1_–d_1_), stem (a_2_–d_2_), rhizome (a_3_–d_3_) and fibrous root (a_4_–d_4_), within 10 days after treatments in 2008. Bars represent standard errors. **, P<0.05; *, P<0.01.

During the first 3 days of experiment 3, fructans in all tissues fell rapidly and remained constant at low level thereafter, except that in the first 0.5 days there was a slight increase in aboveground tissues (Fig. 2a_1_ to 2a_4_). For fructan contents, both clipping and saliva effects (except in leaf) and interactive effects with time (except in stem) were significant. Furthermore in every component tissue, clipped and saliva treated plants had signficantly lower content of fructans, compared to clipped grasses without saliva (Table 3, Fig. 2a_1_ to 2a_4_). This demonstrates that saliva promoted fructans hydrolization in plant tissues. In aboveground tissues, sucrose concentration increased rapidly during the first day of regrowth following treatments, and then it declined rapidly in the following two days. Thereafter, sucrose content did not change significantly in leaf and stem (Fig. 2b_1_ and 2b_2_), whereas in belowground parts, sucrose content increased gradually until the end of the experiment, except that in rhizome it declined 10 days after treatments (Fig. 2b_3_). There was no significant difference between treatments in sucrose (Table 3).

**Table 3:** Repeated measures ANOVA for between-subject effects, treatments (clipping without and with saliva), and within-subject effects (repeated effects), time, and their interaction effects, time×treatments for carbohydrate (fructans, sucrose, glucose and fructose) concentrations of component parts (leaf, stem, rhizome root and fibrous root) of *Leymus chinensis*.

			Fructans		Sucrose		Glucose		Fructose	
		df	*F*	*P*	*F*	*P*	*F*	*P*	*F*	*P*
Leaf	Time	5	110.76	0.0001**	14.07	0.0001**	88.60	0.0001**	23.90	0.0001**
	Treatments	1	2.11	0.1589	1.96	0.1724	6.01	0.0203*	5.21	0.0300*
	Time×Treatments	5	6.73	0.0004**	6.58	0.0003**	0.00	1.0000	0.00	1.0000
Stem	Time	5	28.77	0.0001**	33.01	0.0001**	270.88	0.0001**	82.54	0.0001**
	Treatments	1	6.18	0.0191*	3.33	0.0789	14.52	0.0007**	19.05	0.0002**
	Time×Treatments	5	2.15	0.0882	3.25	0.0201*	0.18	0.9689	0.51	0.7689
Rhizome	Time	5	32.87	0.0001**	15.52	0.0001**	168.76	0.0001	24.02	0.0001**
	Treatments	1	37.60	0.0001**	0.47	0.4992	3.00	0.0954	4.04	0.0550
	Time×Treatments	5	7.48	0.0001**	0.97	0.4576	4.42	0.0051**	1.83	0.1428
Fibrous roots	Time	5	36.16	0.0001**	39.05	0.0001**	153.38	0.0001**	74.88	0.0001**
	Treatments	1	31.89	0.0001**	3.24	0.0822	23.61	0.0001**	55.66	0.0001**
	Time×Treatments	5	6.20	0.0004**	0.84	0.5323	4.78	0.0031**	13.47	0.0001**

**, *P*<0.05;

*, *P*<0.01.

#### Accumulation of glucose and fructose

In the first day of the experiment, glucose content did not change significantly (Fig. 2c_1_ to 2c_4_), except in leaf tissue where glucose decreased by about 50%. During the following period glucose contents increased gradually until the end of the experiment, except that in rhizome it did not significantly change after day 3. In aboveground tissues, fructose contents decreased at the onset of experiment, and increased gradually thereafter (Fig. 2d_1_ and 2d_2_). In rhizome tissue, there was a similar change in fructose content except that fructose began to accumulate after a lag time of 2 days (Fig. 2d_3_). In fibrous root tissue, fructose content of clipped plants with saliva did not vary at the beginning of experiment, increased on day 1 and further after 5 and 10 days (Fig. 2d_4_), whereas in clipped without saliva plants, there was only an increase 3 days after treatments. For monosaccharides, in aboveground tissues difference between treatments was significant, and in belowground parts both treatment effects (except in rhizome) and interactive effects with time (except fructose in rhizome) are significant (Table 3). Clipped and saliva applied plants had slightly more glucose and fructose than clipped plants without saliva (Fig. 2c_1_ to 2d_4_), which suggested that saliva stimulated monosaccharides to accumulate in grasses.

## Discussion

### Saliva effects on tillering

Results from the first two experiments showed that sheep saliva increased the number of tillers, and the number of buds, which could be considered as tillering potential (Fig. 1). The stimulatory effect on tillering is similar to that on branching in woody plants [30], [31], which are both from reserve meristems after the removal of apical dominance due to grazing or clipping [48]. For grasses, vegetative buds and active meristems are of pivotal importance, and successive tiller production by the development of axillary buds allows persistence of perennial grasses [49], [50]. Moreover, increased branching or tillering is one of the main mechanisms of compensatory growth and has been considered as one of mutualistic relationships between grasses and grazers [32], [51], [52]. Before defoliation shoot apex suppresses lateral meristem growth, in which auxins and cytokinins are involved and have opposite effects, that is, auxins inhibit and cytokinins promote branch growth [33], [53]. Herbivory breaks apical dominance and activates reserve meristem to outgrowth, increasing tillers [50]. Since it is known that there is plant growth factors in animal saliva, saliva left on plant during grazing could have a positive effect on plant branching or tillering. Dyer and Bokhari (1976) found that plants experiencing herbivory by grasshoppers were able to produce more tillers than those that were simply clipped, and they suspected that plants were affected by unidentified growth regulators contained in herbivore saliva. Furthermore, they suggested that growth-promoting substance was injected into plant endogenous metabolic process and then translocated to zones of tiller primordium [16]. Therefore, effects of animal saliva on plant growth related to correlation between growth regulators in saliva and meristematic tissue within plant, and are most effective on branching in woody plants or tillering in grass, which was confirmed in Zhang *et al.* (2007) and our results.

In 2006 and 2007, the application of sheep saliva had significantly positive impacts on plant biomass (Fig. 1). This stimulation should be attributed to the increased tillers. Tiller number increased throughout the experiment but most new leaves on these tillers remained unexpanded, and saliva had no effects on height in the two experiments (Table 2). Thus, experimentally induced compensation in biomass was due to an increased number of tillers.

### Saliva effects and clipping intensity

In the first two experiments, saliva effects varied with clipping levels. Specifically, saliva effects were greatest in the 25% and 100% clipping treatments. This effect was especially evident for buds where saliva and clipping had significant interactive effects (Table 2). We believe that these experimental effects are closely associated with the location of meristems within a plant. Herbivory tolerance and compensation often include regrowth by production of new shoots through activation of dormant buds [32]. According to meristem allocation models, the patterns of compensatory regrowth responses following grazing depend on the number of latent meristems that escape from being damaged, and the activation sensitivity of meristems related to the degree of damage [38], [39]. The increased tillers are the result of outgrowth of buds at the base of shoots and along the rhizomes (i.e. the location of the active meristems). The dynamics of tillering is a product of the availability and activity of basal meristems and the hormonal activity of the apical meristems [33].

Animal saliva contains various growth factors [54] and several plant growth regulators, such as cytokinins and auxins, have been found in the salivary systems of insects [23], [24]. These chemicals may be transferred in feeding processes to influence both plants and herbivore [20], [28]. So, saliva addition should be most effective when it is applied near the regions of active cell growth (i.e. meristems) [16], [55], and the magnitude of saliva effects on plant growth should vary with location of herbivory damage. The point of damage in the 25% clipping treatment is up close to the base of apical meristems and young leaves, which exert apical dominance [33], and undoubtedly, and the point of damage in the100% clipping treatment is adjacent to basal meristems. The results demonstrated that it was most effective for saliva to stimulate tillering when applied at the two clipping height levels being closest to either active or basal meristems, and it was shown that, in our results, saliva had the highest positive effects when plants were completely clipped (100%) (Fig. 1). As we hypothesized, the stronger saliva impacts at light clipping intensity validated the expectation that animal saliva played important role in plant compensatory response at light herbivory intensities [13], [14].

### Saliva accelerates carbohydrate mobilization during regrowth

In the third experiment 2008, our results indicated that clipping stimulated the mobilization of fructans. For each part of plant, the significant treatment (or time×treatment) demonstrated that (at least for some of the time) the saliva treated plants were more quickly mobilizing stored fructans. Similarly, glucose and fructose were increasing (Table 3, Fig. 2). This suggested that photosynthesis in the remaining tissues had increased, and the newly produced tissues were photosynthesizing quickly to compensate for the losses to herbivores. Once again the highly significant treatment effects demonstrated that saliva treated plants had a greater compensation response than untreated plants (Table 3, Fig. 2).

Defoliation by grazing or clipping reduces the amounts of the leaf surface and thereby supply and allocation of photosynthate [56]. Consequently carbon supply to aboveground regrowth depends transiently on carbon reserves in the whole seedlings. A plant's ability to rapidly regrow following damage is fundamental to tolerance strategy to herbivory [36], [57]. Soluble carbohydrate reserves are often considered as primary source of carbon for regrowth following defoliation, and rapid mobilization of reserves is crucial. Results in the third trial exhibited that in every component part, fructans were hydrolyzed, and glucose and fructose accumulated after treatments (Fig. 2). This suggests that the whole seedling was a source for resources and supplied carbon for growth of new tissue and production of new tillers. The manner in which resource allocation patterns shift in response to damage is under hormonal control, and auxins may affect bud outgrowth indirectly by mobilizing resource to already differentiated meristems [58], [59]. The impact of saliva on resource mobilization is ascribed to the regulation of various growth factors contained in saliva, which regulate plant growth and metabolism, interacting with regulation by endogenous hormones in plants such as jasmonic acid. Jasmonic acid is one of the products of octadecanoid pathway, which are up-regulated in response to herbivory damage [9]. Interestingly, jasmonates have been shown to have multiple physiological functions, mediating cell expansion and accumulation of sucrose in tuber [11].

### Conclusions

Animal saliva effects on plant growth are much more complex than previously thought. In this study, we found that animal saliva stimulated growth of perennial grasses, accelerating mobilization of photosynthate reserves, enhancing buds tillers and consequently increasing biomass. There were evident physiological responses to saliva application soon after treatments, however, saliva effects on growth properties only occurred one month following treatments. In the present study, we also show that saliva effects varied with clipping levels, stronger at light and complete clipping level. This is associated with meristem distribution within perennial grass, which is adapted to grazing in the long term. The stimulatory effects at light herbivory intensity favour plants to compensate or overcompensate, consistent with the grazing optimization hypothesis. Under intense grazing pressure, saliva contributes to minimize herbivory damage. Saliva effects are beneficial for plants to tolerate continuous herbivory and be adapted to grazing in the long term, which provides insight into the interpretation of mutualism and coevolution between plants and herbivores in grazing systems.
